# Conditional Knockout of IL-1R1 in Endothelial Cells Attenuates Seizures and Neurodegeneration via Inhibiting Neuroinflammation Mediated by Nrf2/HO-1/NLRP3 Signaling in Status Epilepticus Model

**DOI:** 10.1007/s12035-023-03842-6

**Published:** 2023-12-12

**Authors:** Lianlian Wu, Yuhua Zhu, Ying Qin, Honghua Yuan, Lingzhi Zhang, Tianyuan Lu, Quangang Chen, Ankang Hu

**Affiliations:** 1 Experimental Animal Center, Xuzhou Medical University, Xuzhou, 221004 Jiangsu China; 2 Institute of Cellular and Molecular Biology, School of Life Science, Jiangsu Normal University, Xuzhou, 221004 Jiangsu People’s Republic of China

**Keywords:** IL-1R1, Endothelial, Neuroinflammation, Status epilepticus, Nrf2, NLRP3

## Abstract

Studies on the bench and at bedside have demonstrated that the process of epileptogenesis is involved in neuroinflammatory responses. As the receptor of proinflammatory cytokine IL-1β, IL-1β type 1 receptor (IL-1R1) is reported to express abundantly in the endothelial cells in epileptic brains, which is deemed to be implicated in the epileptogenic process. However, whether and how endothelial IL-1R1 modulates neuroinflammatory responses in the pathological process of epileptic seizures and/or status epilepticus (SE) remains obscure. Here, we indicated endothelial IL-1R1 is involved in neuroinflammation, facilitating epilepsy progress via Nrf2/HO-1/NLRP3. In vitro, we observed upregulation of inflammatory cytokines in co-culture model under IL-1β challenge, as well as in BV2 cells after stimulation with conditional medium (CM) from IL-1β-stimulated bEnd.3 cells. In vivo, mice with conditional knockout of endothelial IL-1R1 (IL-1R1-CKO) were generated by hybrid IL-1R1flox/flox mice with Tek-Cre mice. IL-1R1-CKO reduced seizure susceptibility in kainic acid (KA)-induced SE model. In addition, IL-1R1-CKO KA mice exhibited lessened hippocampal neuroinflammation, mitigated neuronal damage, and decreased abnormal neurogenesis. In cognitive behavioral tests, IL-1R1-CKO KA mice presented improvement in learning and memory. Furthermore, we also indicated blockage of endothelial IL-1R1 downregulated the expressions of Nrf2/HO-1/NLRP3 pathway-related proteins. Nrf2-siRNA reversed the downregulation of HO-1, NLRP3, caspase-1, and IL-1β. These results demonstrated CKO of endothelial IL-1R1 reduces seizure susceptibility and attenuates SE-related neurobehavioral damage by suppressing hippocampal neuroinflammation via Nrf2/HO-1/NLRP3.

## Background

Epilepsy is a serious and chronic disease of the central nervous system, associated with significant brain damage produced by SE and characterized by sudden and repetitive onset of seizure, and affects approximately 70 million people worldwide [1, 2]. Epileptic seizures or SE can lead to excitotoxic injury in hippocampus. Despite the availability of effective antiepileptic drugs, around 30% of pharmacoresistant epileptic patients still suffer from epilepsy [3, 4]. Therefore, it is essential to further explore the pathogenesis of epilepsy.

Neuroinflammatory response is one of the vital pathological features of epilepsy. Emerging clinical evidence has validated that neuroinflammation is usually activated in human epileptogenic brain as well as in animal models of epilepsy [5], implying the central roles of neuroinflammation in epileptogenesis. The process of neuroinflammation chiefly involves the activation of microglia and astrocytes, accompanied by the release of numerous inflammation-related cytokines [6, 7]. Increasing evidence indicated that major proinflammatory cytokines, including IL-1β, TNF-α, and IL-6, were upregulated in epileptic patients and animal models of epilepsy [8–12]. It has been established that neuroinflammatory responses contribute to the epileptogenic process, aggravate seizure, and facilitate the generation of proinflammatory cytokines, leading to aberrant neural connectivity and promoted neuronal hyperexcitability as well as decreased seizure threshold [13]. Accordingly, epilepsy and neuroinflammation interact via mutual causation. At present, inhibition of neuroinflammation is deemed as an effective means to alleviate epilepsy, which is supported by clinically positive therapeutic effects of anti-inflammatory drugs on epileptic patients [14, 15]. Moreover, inhibition of hippocampal proinflammatory cytokines can also effectively reduce the frequency of seizures in animal model of epilepsy [16, 17].

IL-1β is a key mediator in response to inflammation, which is sharply increasing in the case of infection, injury, and psychological stress [18]. IL-1β interacts with its specific receptor IL-1R1, implicated in neuroinflammation response in epilepsy pathogenesis. Distribution and function of IL-1R1 have been emphasized earlier due to the pivotal role of IL-1/IL-1R1 signal. Prior studies have shown IL-1R1 exists in almost all cells of innate immune system [19, 20]. However, there has been much dispute regarding the locus of IL-1R1 expression in brain due to the low expression and limitation of technical means. IL-1R1 has been dominantly reported to express in microglia, astrocytes, endothelial cells and neurons. Paradoxically, Liu et al. employed a genetic knockin reporter system in mice to track IL-1R1 and identified that IL-1R1 is primarily expressed in endothelium and barely expressed in astrocytes, but not expressed in microglia under resting state, and another finding is noteworthy that endothelial IL-1R1 could regulate IL-1-induced microglial activation [21]. High expression of IL-1R1 in epileptic brain has been confirmed in human subjects and animal models [22, 23]. There is extensive experimental evidence that blockade of IL-1R1 can reduce epileptic seizure and inflammatory response [14, 24–26]. Nevertheless, these studies mainly concern the blockage of IL-1R1 at the overall level rather than specific cell typology.

Endothelial cells are important components of blood–brain barrier (BBB). The process of epilepsy can incite insult to the BBB. On the grounds that IL-1R1 is primarily expressed by vascular endothelial cells, in this study, we focused on the role of endothelial IL-1R1 in SE. We demonstrated that endothelial IL-1R1 responds to IL-1β to modulate neuroinflammatory responses via microglial activation in cell co-culture model and the knockout mouse model. We identified that endothelial IL-1R1-CKO reduces seizure susceptibility via the inhibition of hippocampal neuroinflammation. Further, we affirmed that endothelial IL-1R1 regulates inflammatory response via Nrf2/HO-1/NLRP3 signaling.

## Methods

### Ethics Statement and Experimental Protocols

**Fig. 1:**
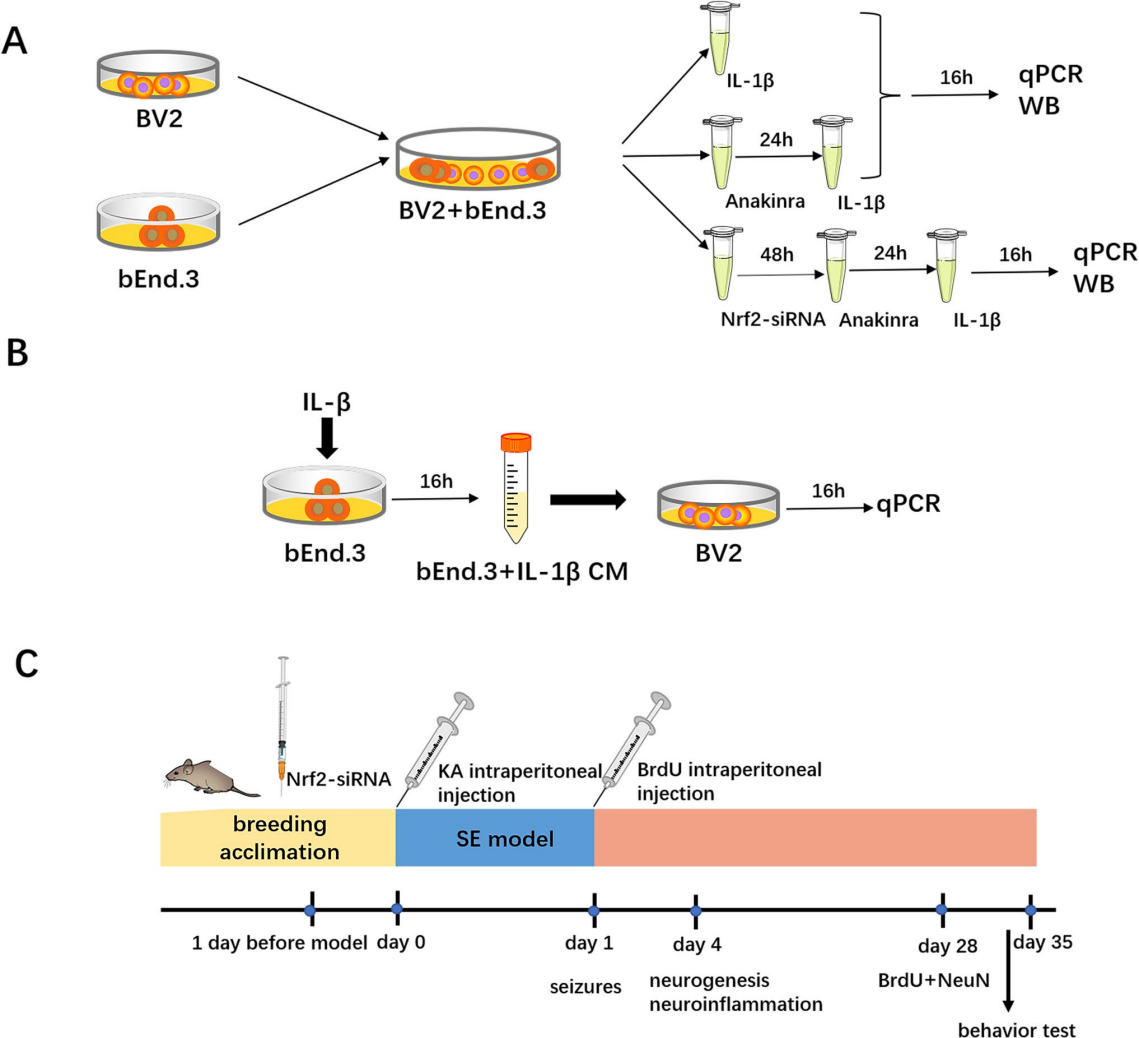
Schematic diagram of the experimental protocols. **A** Expression-level detection of inflammatory factor and protein of co-culture model 16 h after IL-1β stimulation. Nrf2-siRNA incubated with co-culture cells 48 h before IL-1β stimulation. **B** BV-2 cells were treated with conditional medium from IL-1β-stimulated bEnd.3 cells. **C** Drug administration and experimental detection scheme of SE model. Nrf2-siRNA was stereotactically injected in hippocampus 1 day before KA treatment. We detected neurogenesis and neuroinflammation of hippocampus 4 days after KA injection. NeuN-labeled staining was conducted on day 28 after KA treatment. Behavior test was conducted from day 25 to day 32 after KA injection

All experiments were performed in accordance with the guidelines of the European Union and approved by our institutional Animal Care, the Utilization Committee and Procedures for KA-induced epilepsy model and the experiments were approved by the Animal Care and Use Committee at Xuzhou Medical University (Approval number 202207S128, Xuzhou, China). All experiments were conducted in the Experimental Animal Centre, Xuzhou Medical University. All the experimental protocols are shown in Fig. 1.

### Animals

Endothelial IL-1R1 conditional knockout (CKO) mice were generated by hybrid IL-1R1^flox/flox^ mice (C57BL6/JGpt background) with Tek-Cre mice (C57BL6/JGpt background). IL-1R1^flox/flox^ mice and Tek-Cre mice were purchased from Jiangsu Gempharmatech Co., Ltd. (Nanjing, China). The mice were housed in SPF level animal barrier environment with the room temperature (r/t) maintained at 22–25 °C and under a 12-h day-night cycle and ad libitum access to food and water.

### Drugs and Treatment

Recombinant murine IL-1β (Pepro Tech, Suzhou, China) was diluted to a concentration of 10 ng/mL and cells were challenged for 16 h. IL-1R1 inhibitor anakinra (MedChemExpress, Shanghai, China) was diluted in PBS at a final concentration of 1 mg/mL. Cells were incubated with anakinra (100 ng/mL) 24 h before Il-1β treatment. KA (Sigma-Aldrich, USA) was dissolved in saline at a final concentration of 1 mg/mL. Mice were intraperitoneally (i.p.) injected with 25 mg/mL KA to establish epilepsy model. After epilepsy induction, 5-bromo-2′-deoxyuridine (BrdU) (Sigma-Aldrich) dissolved in saline was injected (i.p., 50 mg/kg, twice daily, for 3 consecutive days). Nrf2-siRNA (25, 50, 75 nM, RiboBio, Guangzhou, China) was transfected into cells for 48 h to interfere with Nrf2. In vivo, Nrf2-siRNA (0.25, 0.5, 1 nM) with 1 μL was stereotaxically injected into hippocaumpus (stereotaxic coordinates: AP, − 2.18 mM; ML, 12.50 mm; and DV, 22.00 mm) 24 h before KA injection.

### Cell Lines and Cell Co-culture Model Establishment

The murine brain endothelial cell line bEnd.3 and murine microglial cell line BV-2 were cultured with Dulbecco’s modified Eagle medium (DMEM) containing 10% fetal bovine serum, 100 U/mL penicillin G, and 100 mg/mL streptomycin. Cells were incubated at 37 °C and 5% CO_2_. Cell lines were purchased from Procell Life Science & Technology Co., Ltd. (Wuhan, China). DMEM medium and FBS were purchased from Gibco (Carlsbad, USA).

### KA-Induced SE Model and Administration Procedure

Male mice (8–10 weeks old) were employed to establish animal model and randomly divided into four groups (*n* = 20/group): WT, IL-1R1-CKO, WT KA, and IL-1R1-CKO KA. The mice received 25 mg/kg KA to induce SE. In the control groups, equal dose of saline was injected. Epileptic seizure grade scores were constituted as per the Racine scale [27]: grade 0, no response; grade 1, facial myoclonus; grade 2, head nodding; grade 3, forelimb clonus; grade 4, rearing and severe forelimb clonus; grade 5, rearing, falling, and severe forelimb clonus. Only mice with stages 3–5 of SE were considered eligible and the others were excluded from experiments. Diazepam (10 mg/kg, *i.p*.) was administered 2 h after the onset of seizure to terminate convulsions. The period from KA administration to the initial seizure onset was recorded as onset latency.

### Real-time PCR

The cell sample/dissected hippocampal tissues were homogenized with RNA Easy Fast Tissue/Cell Kit (Tiangen, Beijing, China) to extract total RNA as per the manufacturer’s instructions. Complementary deoxyribonucleic acid (cDNA) was acquired from total RNA using PrimeScript RT reagent kit (Takara, Dalian, China) following the manufacturer’s instructions. The reaction system contained 2 μL cDNA, 0.4 μL forward primer, 0.4 μL reverse primer, and 10 μL SYBR Premix ExTaq II (Takara, Dalian, China). The reaction conditions were 30 cycles of denaturation at 95 °C for 5 s, annealing at 60 °C for 10 s, and then terminated at 95 °C for 15 s. The primers for RT-qPCR were as follows:

- IL-1β F: CACTACAGGCTCCGAGATGAACAAC, R: TGTCGTTGCTTGGTTCTCCTTGTAC
- IL-6 F: ACACATGTTCTCTGGGAAATC, R: AGTGCATCATCGTTGTTCATA
- TNF-α F: GACCCTCACACTCAGATCATCTTCT, R: CCTCCACTTGGTGGTTTGCT

### Western Blotting

The tissue/cell samples were treated with appropriate amount of RIPA lysis buffer (Beyotime, Haimen, China) and protease inhibitor. The tissue samples were fully homogenized by the homogenizer, followed by centrifugation at 14,000 rpm 4 °C for 30 min and supernatant collection. Subsequently, the protein concentration was determined by BCA protein assay kit (Beyotime). The protein samples were prepared to the same concentration and separated by SDS polyacrylamide gel electrophoresis and then transferred to nitrocellulose membrane (NC) (Millipore Corporation, USA). The membranes transferred with protein were blocked with 5% evaporated milk for 2 h at r/t and then incubated with antibodies against mouse anti-IL-1R1 polyclonal antibody (1:500, Invitrogen, CA, USA), rabbit anti-Nrf2 polyclonal antibody (1:1000, Proteintech, Wuhan, China), rabbit anti-HO-1 polyclonal antibody (1:1000, Proteintech), rabbit anti-NLRP3 antibody (1:1000, Abclonal, Wuhan, China), rabbit anti-caspase-1 antibody (1:1000, Abclonal), and rabbit anti-IL-1β antibody (1:500, Abclonal). On the following day, the membranes were rinsed with Tris-buffered saline with Tween-20 (TBST), and then incubated with the secondary antibodies as follows: DyLight 800 goat anti-mouse IgG, DyLight 800 goat anti-rabbit IgG at r/t for 1.5 h. After the membranes were rinsed again, assessment was conducted on an Odyssey scanner (LI-COR, USA), with the greyscale values of bands quantified with ImageJ software (NIH, Bethesda, MD, USA).

### Immunofluorescence Staining

The mice were perfused and fixed with 4% polyformaldehyde. After complete fixation, the brain tissues were dehydrated with 30% sucrose and coronally sliced into 25-μm sections with a freezing microtome. For BrdU immunofluorescence staining, DNA denaturation was performed with 2Ν HCl for 30 min at 37 °C, followed by 0.1 M sodium borate (pH = 8.5) and rinsed in PBS, with identical maneuvers for the other procedures. Slices were permeabilized and blocked with PBS solution containing 1% Triton X-100 and 10% donkey serum for 90 min, followed by incubation with primary antibodies overnight at 4 °C. The primary antibodies included goat anti-IL-1R1 antibody (1:200, R&D systems, MN, USA), rabbit anti-CD31 antibody (1:500, Affinity Biosciences, Cincinnati, OH, USA), rabbit anti-IBA-1 antibody (1:600, Abcam, UK), rabbit anti-CD68 antibody (1:1000, Abcam), rabbit anti-GFAP antibody (1:800, Abcam), rabbit anti-c-Fos antibody (1:1000, Cell Signaling Technology, Danvers, MA, USA), rabbit anti-Nestin antibody (1:500, Abcam, UK), rat anti-BrdU antibody (1:500, Abcam), rabbit anti-Doublecortin (1:500, Abcam), and rabbit anti-NeuN antibody (1:500, Abcam). Subsequently, the slices were rinsed with PBS, followed by incubation with secondary antibodies for 1.5 h at r/t. The secondary antibodies were listed as follows: FITC-conjugated Affinipure Donkey Anti-Goat IgG (1: 500, Proteintech, Wuhan, China), goat anti-rabbit IgG Alexa Fluor 594 conjugate (1:500, ab150080, Abcam, UK), goat anti-rat IgG Alexa Fluor 488 conjugate (1:500, ab150157, Abcam). After wash, the slices were sealed with DAPI Fluoromount-G (Abcam). Finally, the sections were captured and imaged using an IX71 microscope (Olympus, Tokyo, Japan).

### Nissl Staining

Briefly, following isolation and fixation, the brain tissues underwent paraffin embedding and section at 5-μm-thick slides. After dehydration, the slices were treated with Nissl staining solution (Beyotime, Shanghai, China) at 37 °C for 10 min and rinsed with PBS. The sections were then incubated in 70% alcohol differentiation for 5 s, followed by mounting with neutral balsam. The images were photographed with the IX71 microscope (Olympus, Tokyo, Japan). The population of intact neurons in the hippocampal CA3 area was calculated for quantitative analysis with ImageJ software (NIH, Bethesda, MD, USA).

### FJB Staining

Fluoro-Jade B (FJB), a fluorescent dye, can bind with denatured neurons to generate green fluorescence and label the degenerated and necrotic neurons in the nervous tissue. The slices were immersed in a basic ethanol solution comprising 1% sodium hydroxide in 80% ethanol for 5 min. Thereafter, the sections were rinsed for 2 min in 70% ethanol and in distilled water, followed by incubation in 0.06% potassium permanganate solution for 10 min. At the end of wash in distilled water for 1–2 min, the sections were displaced to 0.0004% solution of Fluoro‐Jade B (AmyJet Scientific Inc, Wuhan, China) dissolved in 0.1% acetic acid vehicle followed by immersion of 10 min. Afterwards, the sections were rinsed thrice in distilled water, with each procedure lasting at least 1 min. The slides were dehumidified with paper tissues and air‐dried on a slide heater at 50 °C for at least 5 min. Subsequently, the slides were mounted with coverslips. Digital images were acquired under an IX71 fluorescence microscope (Olympus, Tokyo, Japan).

### Morris Water Maze Test

The Morris water maze (MWM) test was conducted to assess the spatial learning and memory ability of mice. MWM consisted of a circular pool filled with water containing milk to a depth of 30 cm maintained at 20–22 °C and with a platform (10 cm in diameter) below the water surface. The experiment in mice was performed for 5 consecutive days of similar training with the final probe test on the sixth day. Prior to the procedure, the mice were transferred into a water maze without any platforms for adaptive training. In the training session, the mice were allowed for a free swim in the water maze for 60 s. In the case of a mouse failing to reach the platform within 60 s, the mouse was guided to the platform with a stick and was maintained for 15 s and the time spent reaching the platform (escape latency) of each mouse was recorded. With the platform removed on day 6, mice were allowed to swim for 60 s as a probe trial, and the number of crossings of the original site of the platform was recorded. If a mouse had an impaired memory, it would spend shorter in target quadrant and fewer times of crossings.

### Novel Object Recognition Test

The novel object recognition (NOR) test was employed to assess cognitive performance of mice on the grounds that mice with normal cognition should exhibit the tendency to contact novel objects rather than familiar ones. On day 1, each mouse was free to explore the empty arena. On day 2, each mouse was allowed to stay for 5 min in the test chamber containing two familiar objects and was then returned to its cage. On day 3, a novel object of the same shape, size, and color was placed in lieu of a familiar one, and each mouse was allowed for free exploration for 5 min. The durations of each mouse to contact familiar objects (TF) and novel objects (TN) were recorded, with the recognition index calculated as TN/(TF + TN).

### Cell Counting Methods

Five pieces of hippocampal serial sections per animal were selected and underwent immunofluorescence staining, which were analyzed in a blinded manner. The population of positive staining cells in CA1, CA3, or DG regions was manually counted as the image displayed with ImageJ software (NIH, Bethesda, MD, USA) by two independent technicians blinded to the sample grouping. All the data from statistical analysis were then normalized to those in the WT group.

### Statistics

GraphPad Prism software was applied in the statistical analysis. The Shapiro–Wilk test was adopted to determine the normality of the data distribution. Between-group comparisons were performed with Student’s *t*-test (normal distributions) or the Mann–Whitney *U* test (non-normal distributions). Multiple-group analyses were conducted with one- or two-way ANOVA followed by a Tukey post hoc test for normal distributions. Experimental data are presented as the mean ± standard deviation (SD), with *p* value < 0.05 considered to be statistically significant.

## Results

### IL-1β Challenge of Endothelial IL-1R1 to Induce Inflammation

**Fig. 2:**
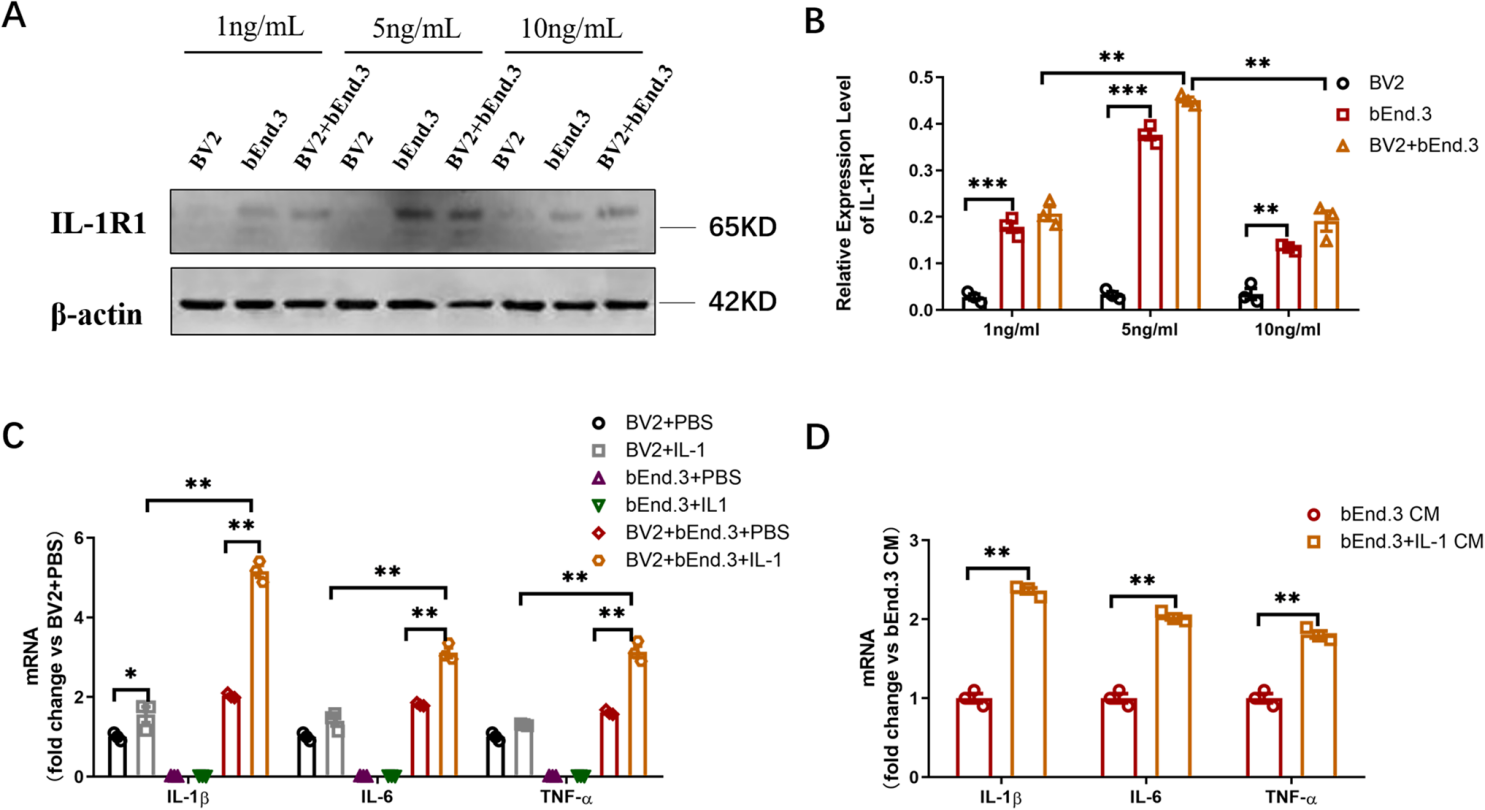
IL-1β directly challenged endothelial IL-1R1 to induce inflammation. **A** IL-1R1 protein level of BV2 cells, bEnd.3 cells, and co-culture cells under different concentrations of IL-1β stimulation detected by western blotting. **B** Bar graphs of relative optical density of IL-1R1 expression. **C** The mRNA expression of IL-1β, IL-6, and TNF-α of BV2 cells, bEnd.3 cells, and co-culture cells under IL-1β stimulation. **D** The mRNA expression of IL-1β, IL-6, and TNF-α of bEnd.3 cells and CM-stimulated bEnd.3 cells. Data are expressed as mean ± SD; *n* = 3/group, *p < 0.05, **p < 0.01, ***p < 0.001

IL-1R1 mRNA is reported to be comparatively overexpressed in endothelial cells [21], with microglia being the main producers of inflammatory factors. Accordingly, an in vitro cell co-culture model was employed to test the action scheme of the IL-1β challenge-induced inflammation. With the establishment of optimal concentration of IL-1β to induce inflammation, western blotting was conducted. IL-1R1 protein barely expressed in microglial cell line (BV-2) cells, but largely expressed in brain endothelial cell line (bEnd.3). IL-1R1 level was at the peak at the IL-1β concentration of 5 ng/mL (Fig. 2A, B), thereby designated for subsequent experiments. mRNA levels of inflammatory factors (IL-1β, IL-6, TNF-α) were detected by qPCR, which revealed modest upregulation in BV2 cells with IL-1β challenge in contrast to significant upregulation in the co-cultured cells (BV2 and bEnd.3) (*p* < 0.01, Fig. 2C). Nevertheless, in the absence of IL-1β challenge, the mRNA levels of inflammatory factors were markedly reduced in the co-cultured cell model. Next, mRNA expression levels of inflammatory factors were tested in BV2 cells treated with conditional medium, which revealed significant upregulation (*p* < 0.01, Fig. 2D). These findings suggested the central role of endothelial IL-1R1 in inflammation production, which could be attributed to direct association with endothelial IL-1R1 rather than microglial IL-1R1.

### Endothelial IL-1R1-CKO Reduced Seizure Susceptibility in KA-Treated Mouse Model

**Fig. 3:**
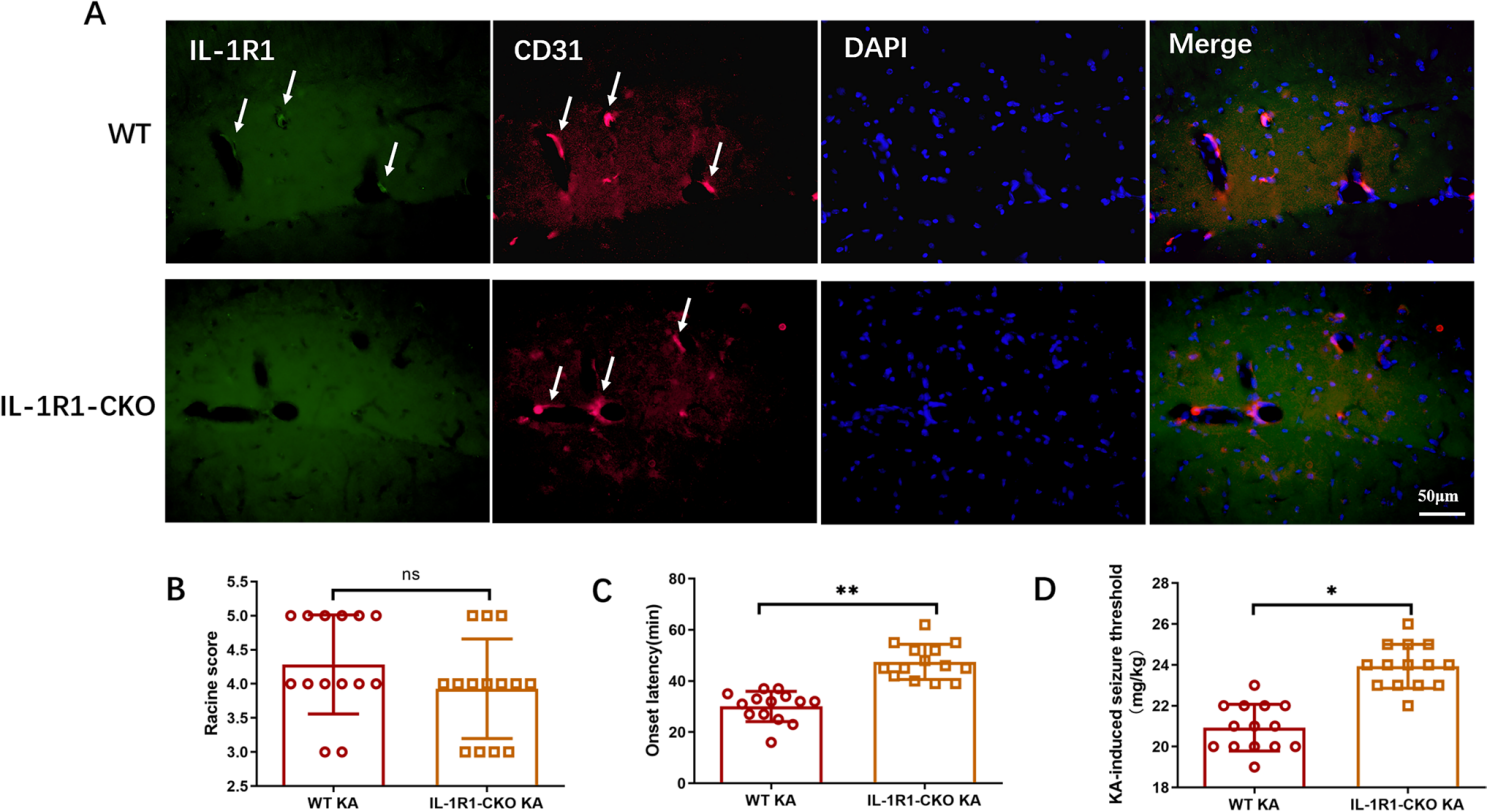
Endothelial IL-1R1-CKO reduced seizure susceptibility in KA-induced SE mice. **A** Immunofluorescence double labeling of IL-1R1 and CD31 in WT mice and IL-1R1-CKO mice, showing IL-1R1 complete knockout in endothelial cells. **B** Comparison of Racine score between WT KA mice and IL-1R1-CKO KA mice. **C** Comparison of onset latency between WT KA mice and IL-1R1-CKO KA mice. **D** Comparison of KA-induced seizure threshold between WT KA mice and IL-1R1-CKO KA mice. Data are expressed as mean ± SD;* n* = 14/group, *p < 0.05, **p < 0.01

Endothelial IL-1R1-CKO mice were developed by crossbreeding IL-1R1^flox/flox^ mice with Tek-Cre mice. Double immunofluorescence labeling of CD31/IL-1R1 was employed to confirm complete ablation of IL-1R1 in endothelial cells (Fig. 3A). The mice were observed for 2 h as from the initial seizure onset after KA treatment, with the behavior profiles recorded. Consequently, no marked difference in Racine scores was observed between the endothelial IL-1R1-CKO KA group and WT KA group (Fig. 3B). Endothelial IL-1R1-CKO in mice markedly prolonged the onset latency versus the WT group (Fig. 3C). Moreover, minimum concentration of KA to induce seizure in the endothelial IL-1R1-CKO KA group was higher than that in the WT KA group (Fig. 3D). The results indicated genetic deletion of IL-1R1 in endothelial cells may reduce seizure susceptibility with KA treatment.

### Endothelial IL-1R1-CKO Decreased Hippocampal Inflammation in KA-Induced SE Mouse Model

**Fig. 4:**
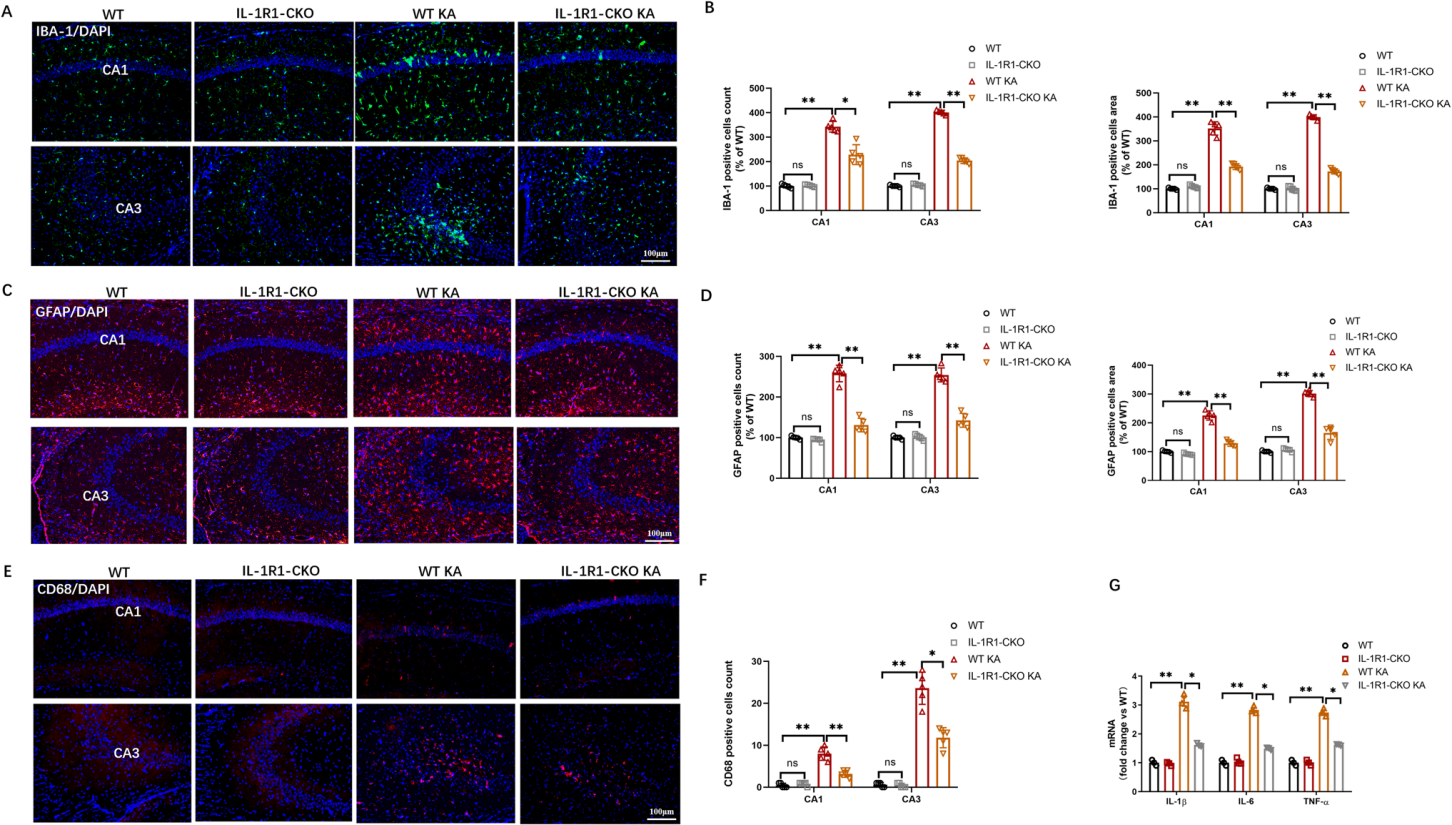
Endothelial IL-1R1-CKO decreases hippocampal inflammation in SE mice. **A** Representative images of immunofluorescence staining of IBA-1 in the hippocampal CA1 and CA3 regions. **B** Bar chart for the number of IBA-1-positive cells and fluorescence area of IBA-1-positive cells as percentage of WT. **C** Representative images of immunofluorescence staining of GFAP in the hippocampal CA1 and CA3 regions. **D** Bar chart for the number of GFAP-positive cells and fluorescence area of GFAP-positive cells as percentage of WT. **E** Representative images of immunofluorescence staining of CD68 in the hippocampal CA1 and CA3 regions. **F** Graph for the count of CD68-positive cells. **G** The mRNA level of IL-1β, IL-6, and TNF-1α in the hippocampus in WT, IL-1R1-CKO, WT KA, and IL-1R1-CKO KA mice. Data are expressed as mean ± SD; *n* = 5/group, *p < 0.05, **p < 0.01

Endothelial IL-1R1 is reportedly involved in glial activation [21]. Accordingly, we explored whether IL-1R1 deletion in endothelial cells can reduce neuroinflammatory reaction in mice following SE. We detected hippocampal inflammation by labeling microglia with IBA-1 and CD68 and labeling astrocytes with GFAP via immunofluorescence staining on day 3 after SE induction. The mRNA levels of inflammatory cytokines in hippocampus were detected by qPCR. In effect, the populations and areas of IBA-1 + and GFAP + cells were significantly increased in CA1 and CA3 areas in epileptic hippocampus, indicating the activation of microglia and astrocytes after KA induction of epilepsy. However, endothelial IL-1R1-CKO reversed these that we observed obviously decreased populations and areas of IBA-1 + and GFAP + cells compared with the WT KA group (Fig. 4A–D). Similarly, immunofluorescence also confirmed that endothelial IL-1R1-CKO also reduced the populations of D68 + cells in comparison with the WT KA group (Fig. 4E, F). Activated glia are the main source of inflammatory cytokines, with mRNA level of inflammatory cytokines upregulated in hippocampus. However, KA induction in endothelial IL-1R1-CKO mice significantly reduced inflammatory responses, with less release of inflammatory cytokines in comparison with the WT KA group (Fig. 4G). Taken together, these findings demonstrated endothelial IL-1R1-CKO can relieve hippocampal inflammation in KA-induced SE mouse model.

### Endothelial IL-1R1-CKO Inhibited Neuronal Damage and Neuronal Activity in KA-Induced SE Mice

**Fig. 5:**
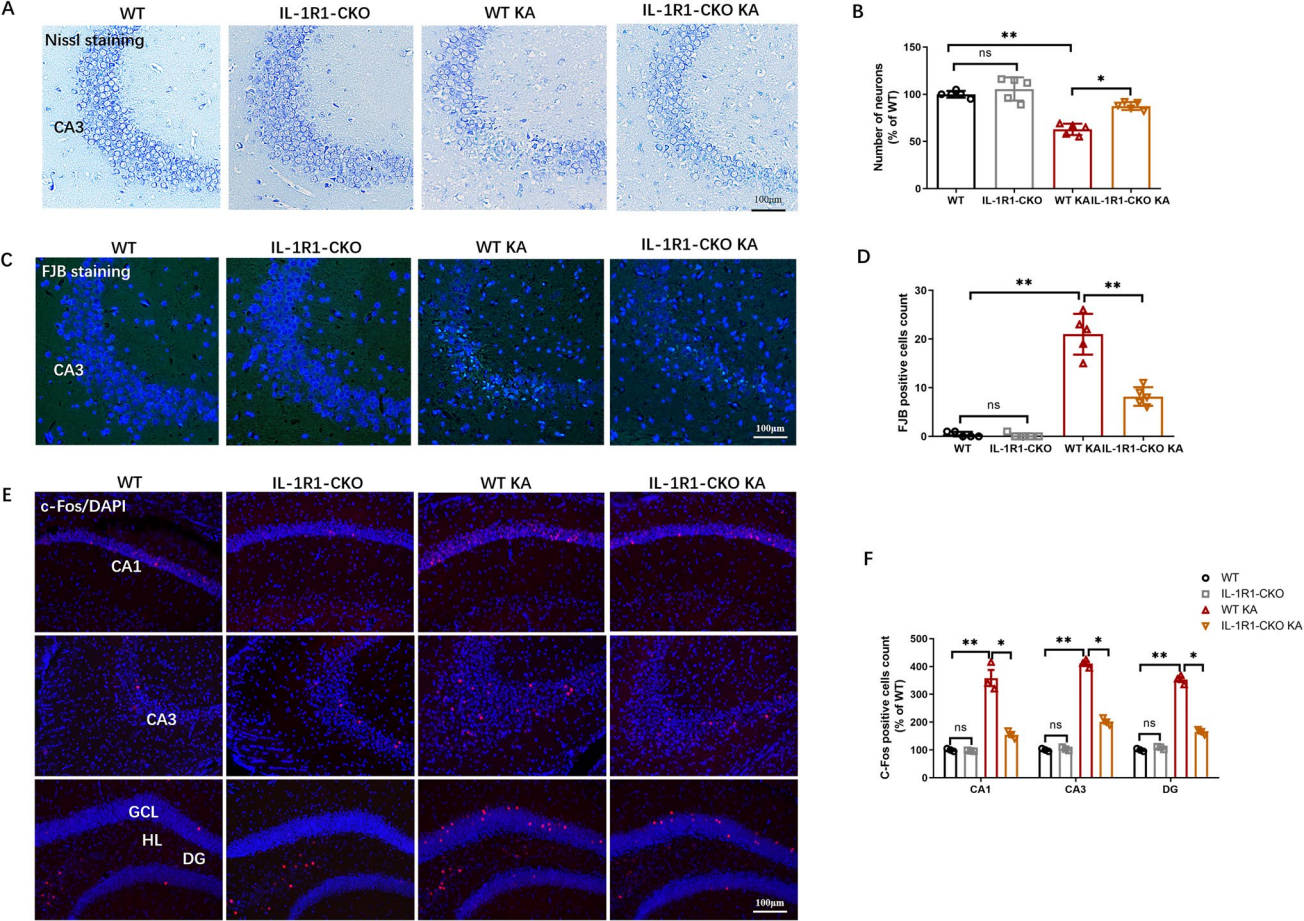
Endothelial IL-1R1-CKO inhibits neuronal damage and neuronal excitability in SE mice. **A** Representative images of Nissl staining of hippocampal CA3 region. **B** Bar chart of the number of neurons in Nissl staining result as percentage of WT. **C** Representative images of FJB staining of hippocampal CA3 region. **D** Statistical histogram of the number of FJB-positive cells. **E** Immunofluorescence staining of c-Fos in CA1, CA3, and DG regions of hippocampus. **F** Graph of the number of c-Fos-positive cells as percentage of WT. Data are expressed as mean ± SD; *n* = 5/group, *p < 0.05, **p < 0.01

To assess neuronal damage in hippocampus following SE, Nissl staining was conducted to estimate neuronal loss and FJB staining was employed for neuronal degeneration. Nissl staining showed a significant neuronal loss in CA3 region in the WT KA group compared to the WT group, with this reduction evidently in endothelial IL-1R1-CKO mice than in WT KA mice (Fig. 5A, B). The population of FJB + neurons was observed to markedly increase in CA3 region after KA induction, while deletion of endothelial IL-1R1 significantly diminished the number of FJB + neurons in KA-induced SE model (Fig. 5C, D). In addition, c-Fos fluorescent staining was performed to assess neuronal activity. In CA1, CA3, and DG regions, epilepsy precipitated marked rise of c-Fos + cell counts. In endothelial IL-1R1-CKO KA mice, the number of c-Fos + cells significantly declined after epilepsy modeling when compared to the WT KA group (Fig. 5E, F), showing deletion of endothelial IL-1R1 may contribute to the decline of neuronal activation. These results indicated endothelial IL-1R1-CKO can prevent neuronal damage and inhibit neuronal activation in mice following SE.

### Endothelial IL-1R1-CKO Repressed Abnormal Neurogenesis in Hippocampus in KA-Induced Mouse Model of SE

**Fig. 6:**
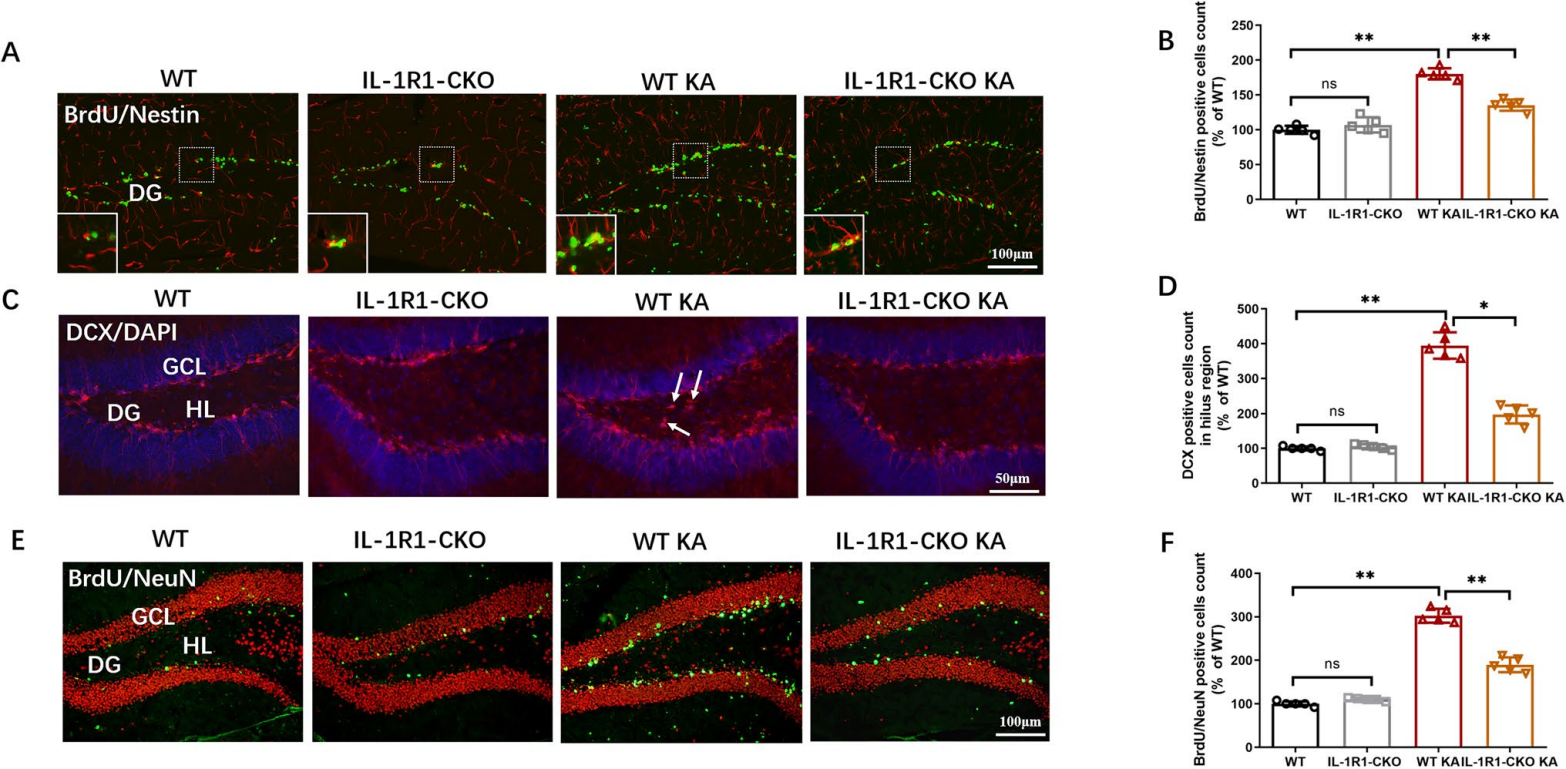
Endothelial IL-1R1-CKO repressed abnormal neurogenesis of hippocampus in SE mice. **A** Representative images of immunofluorescence labeling of BrdU/Nestin in hippocampal DG region. **B** Bar chart showing the count of BrdU/Nestin-positive cells as percentage of WT. **C** Representative images of immunofluorescence labeling of DCX in hippocampal DG region. **D** Graph showing the number of DCX-positive cells in the hilus region as percentage of WT. **E** Representative images of immunofluorescence labeling of BrdU/NeuN in hippocampal DG region. **F** Quantification of BrdU/NeuN-positive cells as percentage of WT. Data are expressed as mean ± SD; *n* = 5/group, *p < 0.05, **p < 0.01

Rapid proliferation of neural progenitor cells and the ectopic migration of newborn neurons in hippocampus were induced at the initial stages of epileptogenesis [28–30]. Here, co-labeling of BrdU and Nestin was employed to visualize the proliferation of neural progenitor cells. The quantity of BrdU/Nestin-positive cells was evidently enhanced 3 days after KA administration. Conditional deletion of endothelial IL-1R1 substantially blunted the SE-induced proliferation of neural progenitor cells (Fig. 6A, B). KA treatment also increased DCX + cells in the hilus of hippocampus, indicative of ectopic migration of newborn neurons, whereas the count of DCX-positive cells in the hilus was significantly decreased in endothelial IL-1R1-CKO KA mice compared to WT KA mice (Fig. 6C, D). To profile the survival of newborn neurons in epileptic hippocampus, we detected the number of BrdU/NeuN + cells at 4 weeks after KA injection. The results showed that viable neurons were mainly located in subgranular zone (SGZ) of DG and the quantity of BrdU/NeuN + cells was notably increased in the SGZ region of epileptic hippocampus, which indicated the fate of newborn neurons resulted from acute epilepsy. A significant decrease of BrdU/NeuN + cells was observed in the endothelial IL-1R1-CKO group versus the WT group of SE hippocampus (Fig. 6E, F). These results indicated IL-1R1 deletion in endothelial cells suppressed abnormal neurogenesis of hippocampus in KA-induced SE model.

### Endothelial IL-1R1-CKO Ameliorated Cognitive Impairments in KA-Induced Mouse Model of SE

**Fig. 7:**
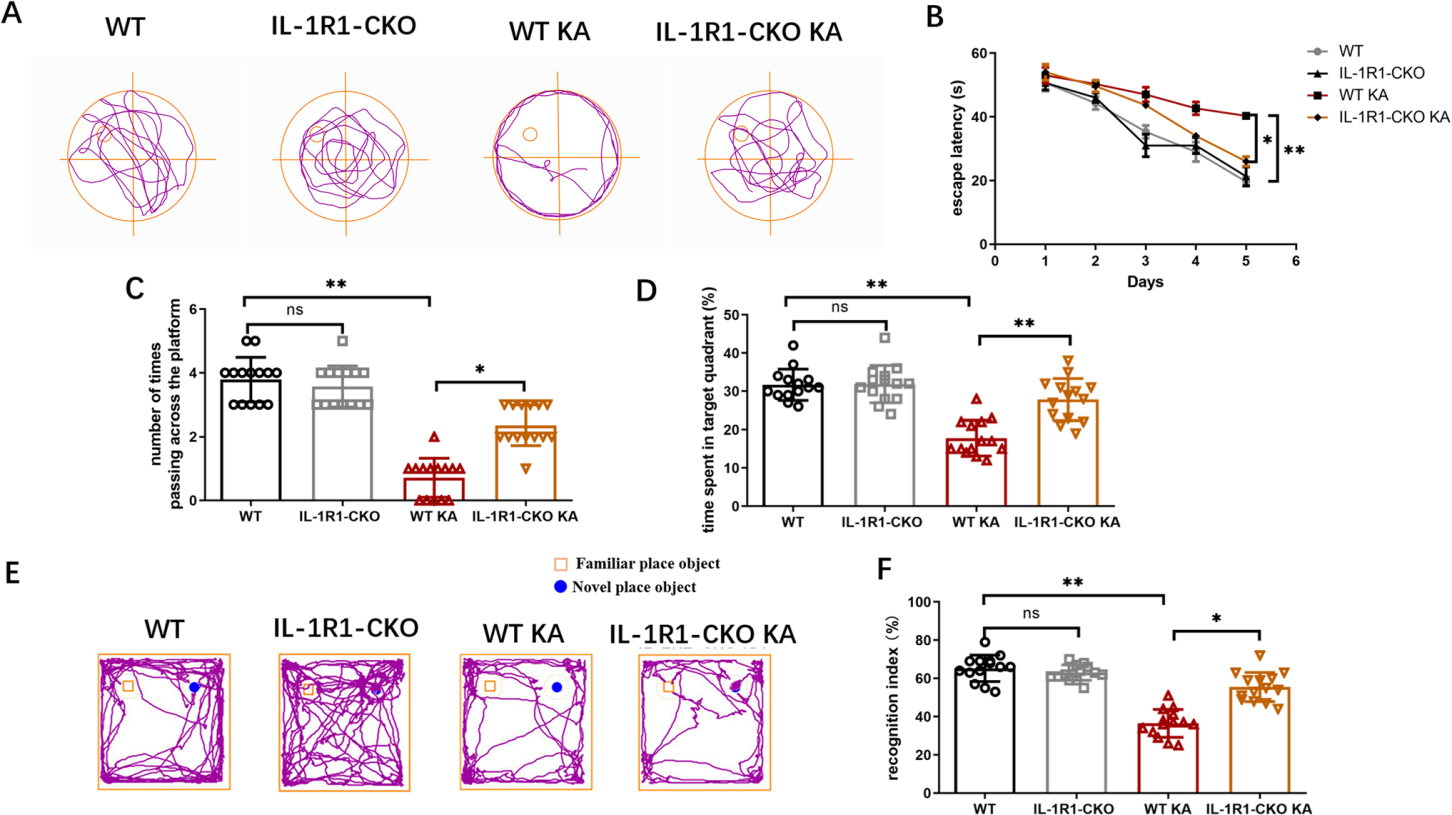
The effect of endothelial IL-1R1-CKO on cognition in SE mice. **A** The representative swim paths of mice on the test day. **B** The time until finding the platform was recorded as escape latency in the training period (days 1–5). **C** The number of times of crossing the platform (day 6). **D** The time spent in the target quadrant (day 6). **E** Representative locomotion tracking of mice in the NOR test. **F** Comparisons of cognition index in each group. Data are expressed as mean ± SD; *n* = 14/group, *p < *0.05*, **p < 0.01

To determine whether SE affect cognitive function, the Morris water maze and novel object recognition were performed 4 weeks after KA treatment. Before a final probe test, spatial acquisition trials were conducted for 5 consecutive days. The representative swim paths of mice on the test day are showed in Fig. 7A. Escape latency was significantly longer after SE induction, suggesting a weaker learning ability. In our study, endothelial IL-1R1-CKO KA mice spent shorter time to find the platform than WT KA mice on day 5 (Fig. 7B). In the final testing phase, with the platform removed from the target platform, fewer times of crossing the platform were observed in the KA group and increasing times in the endothelial IL-1R1-CKO KA group compared with the WT KA group (Fig. 7C). Additionally, the percentage of time swimming in the target quadrant was higher in the endothelial IL-1R1-CKO KA group than in the WT KA group (Fig. 7D). This result in the Morris water maze test may indicate weakened impaired memory in the IL-1R1-CKO KA group than in the WT KA group. Representative locomotion tracking of mice in the NOR test is showed in Fig. 7E. In the novel object recognition test, we observed KA-treated mice spent less time interacting with the novel object and the recognition index was lower. Endothelial IL-1R1-CKO KA mice were more willing to contact novel objects and spent more time interacting with the novel objects when compared with WT KA mice. The recognition index was significantly increased in the endothelial IL-1R1-CKO KA group compared with the WT KA group (Fig. 7F). Our findings suggested endothelial IL-1R1-CKO could rescue impaired learning and spatial memory in SE mice.

### Blockage of Endothelial IL-1R1 May Inhibit Neuroinflammation Through Nrf2/HO-1/Caspase-1 Pathway

**Fig. 8:**
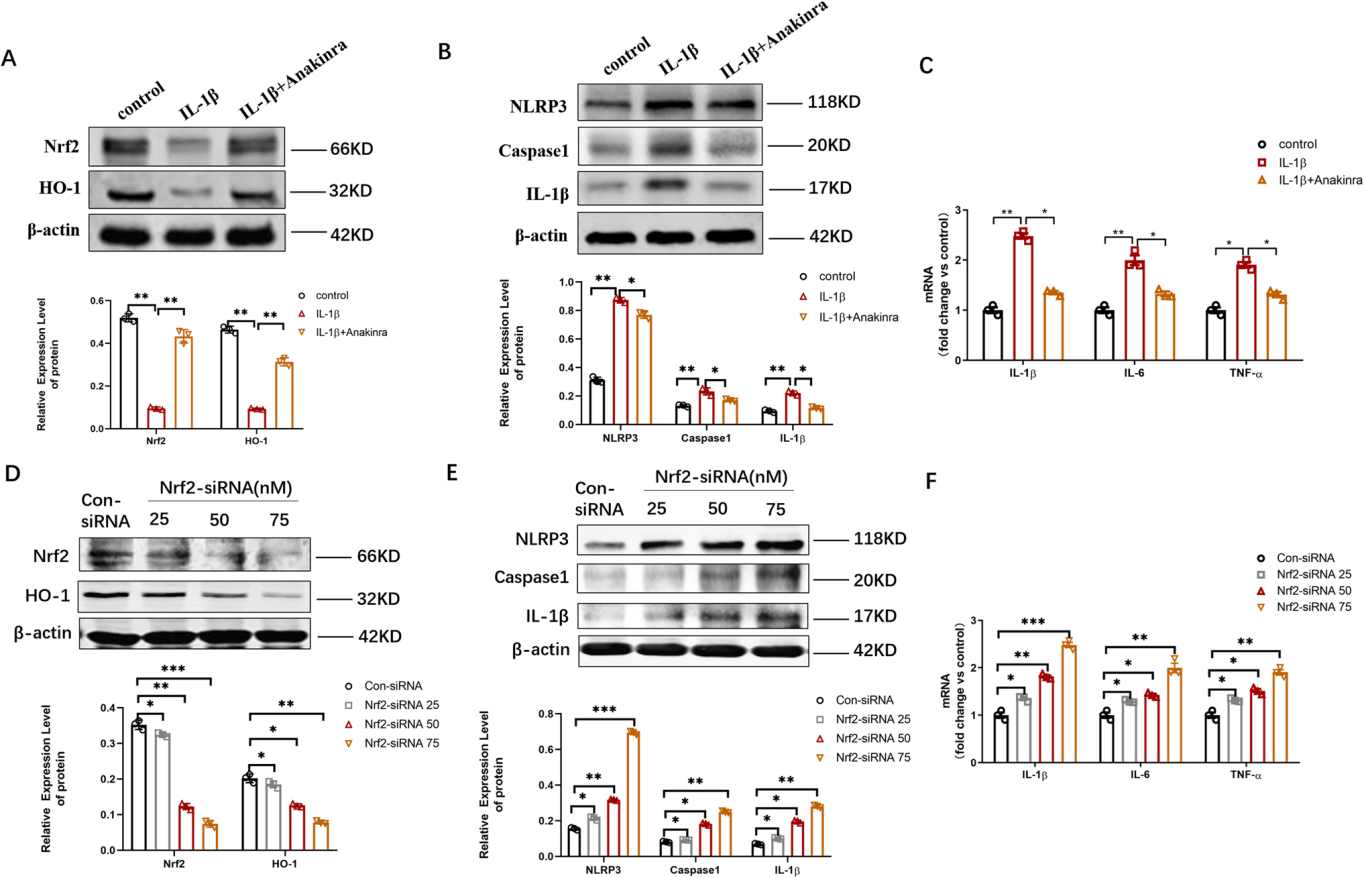
In vitro, endothelial IL-1R1 inhibited neuroinflammation via Nrf2/HO-1/caspase-1 pathway. **A** Western blotting analysis showing the expression level of Nrf2 and HO-1 of co-culture model with IL-1R1 blockage. **B** Western blotting analysis showing the expression level of NLRP3, caspase-1, and IL-1β of co-culture model with IL-1R1 blockage. **C** The mRNA expression of IL-1β, IL-6, and TNF-α in control, IL-1β, and IL-1β + Anakinra groups. **D** The expression level of Nrf2 and HO-1, when Nrf2 was interfered with different dosing of Nrf2-siRNA, as detected by western blotting. **E** The expression level of NLRP3, caspase-1, and IL-1β, when Nrf2 was interfered with different dosing of Nrf2-siRNA, as detected by western blotting. **F** The mRNA expression of IL-1β, IL-6, and TNF-α in control, con-siRNA, and Nrf2-siRNA groups. Data are expressed as mean ± SD; *n* = 3/group, *p < 0.05, **p < 0.01

**Fig. 9:**
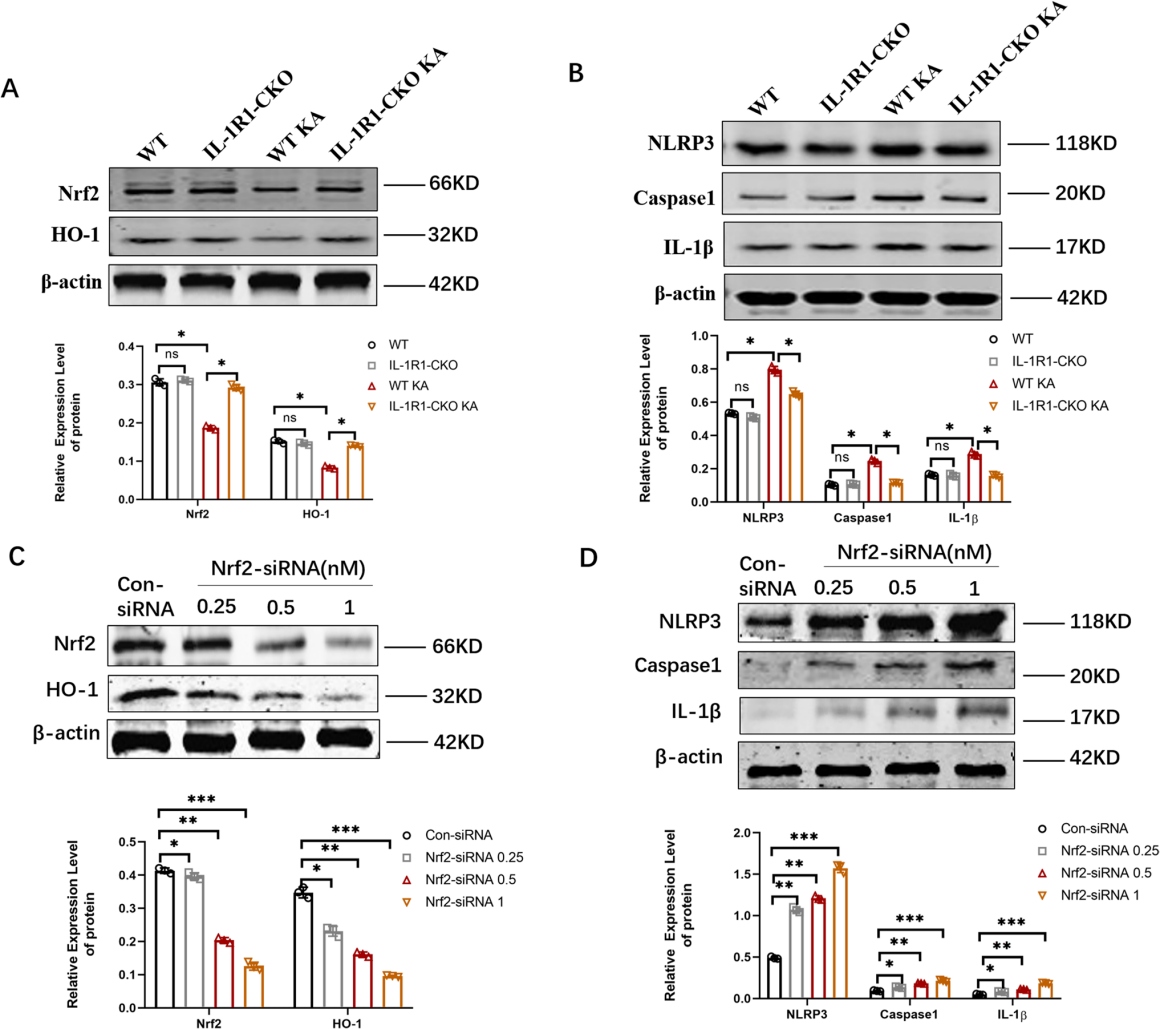
In vivo endothelial IL-1R1 knockout inhibited neuroinflammation via Nrf2/HO-1/caspase-1 pathway. **A** Western blotting analysis of the expression level of Nrf2 and HO-1 in WT, IL-1R1-CKO, WT KA, and IL-1R1-CKO KA mice. **B** Western blotting analysis of the expression level of NLRP3, caspase-1, and IL-1β in WT, IL-1R1-CKO, WT KA, and IL-1R1-CKO KA mice.** C** Western blotting profiling the expression level of Nrf2 and HO-1 with different dosing of Nrf2-siRNA injection. **D** Western blotting profiling the expression level of NLRP3, caspase-1, and IL-1β with different dosing of Nrf2-siRNA injection. Data are expressed as mean ± SD; *n* = 3/group, *p < 0.05, **p < 0.01

To determine the signaling pathway involved in inflammation by endothelial IL-1R1, western blotting was performed. In cell co-culture model, the expression level of Nrf2 and HO-1 was prominently upregulated and the level of NLRP3, caspase-1, and IL-1β significantly downregulated when IL-1R1 was inhibited by anakinra (Fig. 8A, B). Moreover, mRNA levels of inflammatory factors were detected by qPCR. Inhibition of IL-1R1 drastically decreased the mRNA levels of inflammatory factors (Fig. 8C). During the blockage of Nrf2 with different dosing of Nrf2-siRNA (25, 50, 75 nM), HO-1 level was decreased, whereas the levels of NLRP3, caspase-1, and IL-1β were enhanced as well as mRNA levels of inflammatory factors (Fig. 8D–F), which illustrated gradient changes. In epileptic mouse model, we observed the expression of Nrf2 and HO-1 was downregulated and that of NLRP3, caspase-1, and IL-1β levels upregulated in epileptic hippocampus. Nevertheless, endothelial IL-1R1-CKO reversed the variation in protein expression (Fig. 9A, B). Nrf2-siRNA (0.25, 0.5, 1.0 nM) was stereotactically injected in hippocampus of endothelial IL-1R1-CKO KA mice, with the gradient variation in protein expression consistent with that in cell co-culture model (Fig. 9C, D). The above results suggested endothelial IL-1R1-CKO inhibited neuroinflammation presumably via Nrf2/HO-1/caspase-1 pathway.

## Discussion

This current experiment is based on our previous study which demonstrated IL-1R1 expression was upregulated in epileptic hippocampus and LPS-induced peripheral inflammation aggravated seizure activity through IL-1R1 signaling [31], emphasizing the implication of IL-1R1 in peripheral-central inflammation. Despite the controversy, endothelial cells are the most abundant sites with IL-1R1 expression in the center nervous system according to the report. Many researchers are interested in the function of endothelial IL-1R1 in central nervous system diseases. As reported by Judith Hauptmann et al., IL-1R1 deletion in endothelial cells obviously alleviated the severity of autoimmune encephalomyelitis [32]. However, less is known about endothelial IL-1R1 in epileptogenesis. We hypothesized endothelial IL-1R1 serves as a pivotal mediator inducing neuroinflammation in the progression of epilepsy. Accordingly, BV2-bEnd.3 cell co-culture model and a transgenic mouse with conditional knockout of IL-1R1 in endothelial cells were employed in our study, and we established SE models by intraperitoneal injection of KA.

IL-1β is generally reported to directly act on microglial IL-1R1 to expand inflammation in the brain. Here, we identified bEnd.3 cells are indispensable for the production of abundant inflammatory factors in BV2 cells challenged with IL-1β. Intriguingly, IL-1β directly acted on endothelial cells, which was a prerequisite to the production of inflammatory factors from BV2 cells. With endothelial IL-1R1 blocked with anakinra, a significant decrease of inflammatory factors was observed. Consistent with our results, Ling Zhu et al. reported IL-1-induced microglial proinflammatory cytokine by indirect activation of endothelial IL-1R1 rather than microglia [33], plausibly reinforcing the key role of endothelial IL-1R1 in amplifying neuroinflammation.

There is an array of evidence that IL-1β/IL-1R1 is correlated with epilepsy severity. It is reported IL-1RA, an endogenous antagonist of IL-1R1, either overexpressed or exogenously injected, can suppress seizures [34]. A research on animals suggested that IL-1β treatment does not increase the susceptibility to seizure in epileptic mice in the case of IL-1R1 knockout [35]. Fortunately, in our research, endothelial IL-1R1-CKO in mice prolonged onset latency, but did not affect seizure grade. We also identified higher threshold of KA-induced seizure in CKO group mice, signifying the difficulty in kindling the CKO mice. Our findings validated the involvement of endothelial IL-1R1 in SE. Recently, a similar study by Qiming Li et al. described a mouse model with endothelial IL-1R1 knockdown displayed a decrease in incidence and severity of encephalomyelitis [36]. Here, we focused on endothelial IL-1R1 in SE model for the first time.

It is well established neuroinflammation is commonly activated in epileptic brain, characterized by activation of microglia and astrocytes and secretion of various types of proinflammatory cytokines. The hippocampal CA1 and CA3 areas are commonly deemed as sensitive areas associated with acute inflammation under different brain insults [37]. Here, we affirmed a significant increase of the quantity and area of IBA-1- and GFAP-labeled cells in hippocampal CA1 and CA3 regions in KA-induced mice, with similar variations in the mRNA levels of proinflammatory cytokines. Meanwhile, we identified this process was reversed in IL-1R1-CKO mice. Our results showed blockage of endothelial IL-1R1 significantly inhibited hippocampal inflammation after SE establishment, implying the considerable implication of endothelial IL-1R1 in inflammation response after the onset of epileptic seizure. As ES Wohleb et al. reported, knockdown of endothelial IL-1R1 attenuated stress-induced neuroinflammation [38].

Neuronal death and aberrant neurogenesis are deemed as typical pathological characteristics in hippocampus of epileptic brain, which manifests as cognitive and behavioral impairments [39]. It is reported that excessive neuroinflammation tends to enhance neuronal excitability, leading to neuronal loss and death [40]. It may also contribute to hippocampal abnormal neurogenesis. In fact, epileptic seizure always triggers aberrant neurogenesis. In this study, we recognized substantial neuronal loss or death in hippocampal CA3 region after SE induction, with prominently increased count of c-Fos-labeled seizure-activated neurons in CA1, CA3, and DG areas. These injuries appeared to be mitigated in endothelial IL-1R1-CKO mice in which neuronal loss and activation apparently decreased. In addition, we also observed additive progenitor cell proliferation until 4 weeks and obvious ectopic neurogenesis in KA-induced SE model. These alterations were reversed in endothelial IL-1R1-CKO group when induced with KA. This trend seems to be consistent with the inflammation of the hippocampus. Even a single episode of seizures can lead to profound pathological damage in hippocampus [41]. Our finding indicated specific target to endothelial cell IL-1R1 may rescue the pathological change. Neuroinflammation seems not only as an inducer of neurogenesis [42], but also as a detrimental cause of neuronal hyperexcitability in epileptogenesis.

Hippocampal damage caused by SE can bring about cognitive impairment [43]. Correspondingly, several animal models of TLE exhibit memory dysfunction, especially spatial memory impairment [44]. According to the research by Kyung-Ok Cho et al., ablation of aberrant hippocampal neurogenesis can normalize epilepsy-associated cognitive deficits [45], suggesting the connection between neurogenesis and cognition. Here, we performed behavioral tests to examine cognitive impairment, with the finding that SE mice showed significant cognitive decline, while deletion of endothelial IL-1R1 alleviated impaired spatial memory. The various mechanisms of disease occurrence are not independent. In effect, there is a close interconnection between neuroinflammation, neurogenesis, neuronal hyperexcitability, and cognitive function in epileptic brain.

Based on the above results, we concluded that IL-1R1 knockout in endothelial cells may mitigate epileptic seizures and SE-related cognitive impairment by suppression of neuroinflammation. Further, we explored the molecular pathway with the result that endothelial IL-1R1 can promote neuroinflammation via the Nrf2/HO-1/NLRP3 pathway. Nuclear factor erythroid 2-like (Nrf2) is a key regulatory factor of endogenously induced defense system, and recently, its anti-inflammatory effect is attracting attention. Recent researches demonstrated Nrf2 activation can attenuate the persistent neuroinflammatory response and protect adjacent neurons from LPS-induced microglial activation [46]. The antioxidant enzyme heme oxygenase-1 (HO-1) expression is strictly regulated by Nrf2 and reportedly produces major anti-inflammatory effects in the endothelial cells [47]. Increasing studies focused on anti-inflammatory function of the Nrf2/HO-1 signaling pathway. Of note, recent studies identified that IL-1 signaling suppressed the expression of HO-1 [32, 48]. We thus postulated that endothelial IL-1R1 regulated inflammation response via Nrf2/HO-1. Our results showed blockage of endothelial IL-1R1 can upregulate expression of Nrf2 and HO-1 and downregulate the protein expression of NLRP3, caspase-1, and IL-1β in SE hippocampus. Nrf2-siRNA can reverse downstream protein expression and enhance the levels of inflammation factors, which founded our supposition. Our findings strongly authenticated the implication of regulation of Nrf2/HO-1/NLRP3 signaling pathway in neuroinflammation in this context. Ling Zhu et al*.* reported IL-1 indirectly induced microglial proinflammatory cytokine by activation of endothelial IL-1R1 rather than microglia. They further identified a factor after direct action on endothelial cells which they considered to be a thermostable molecule of a size larger than 50 kDa [33]. Accordingly, we speculated the activated Nrf2/HO-1/NLRP3 signal may induce intermediate factor production to activate microglia, inducing inflammation, which needs further study.

## Conclusions

In conclusion, our findings emphasized the critical role of endothelial IL-1R1 in mediating pathogenesis in epilepsy. Deletion of endothelial IL-1R1 can reduce seizure susceptibility and alleviate SE-related neurobehavioral damage, maybe alleviating hippocampal inflammation via regulation of Nrf2/HO-1/NLRP3 signal axis.

## Data Availability

All the necessary data are included within the current study. Further data will be shared by request.

## Abbreviations

BBB: Blood-brain barrier
CKO: Conditional knockout
CM: Conditional medium;
DG: Dentate gyrus
HO-1: Heme oxygenase-1
KA: Kainic acid
IL-1R1: IL-1β type 1 receptor
Nrf2: Nuclear factor erythroid 2-like
SGZ: Subgranular zone
